# Examining the Supports and Advice That Women With Intimate Partner Violence Experience Received in Online Health Communities: Text Mining Approach

**DOI:** 10.2196/48607

**Published:** 2023-10-09

**Authors:** Vivian Hui, Malavika Eby, Rose Eva Constantino, Heeyoung Lee, Jamie Zelazny, Judy C Chang, Daqing He, Young Ji Lee

**Affiliations:** 1 Center for Smart Health, School of Nursing The Hong Kong Polytechnic University Hong Kong China (Hong Kong); 2 Health and Community Systems, School of Nursing University of Pittsburgh Pittsburgh, PA United States; 3 Department of Psychology, Swarthmore College Swarthmore, PA United States; 4 Department of Obstetrics, Gynecology & Reproductive Sciences, and Internal Medicine University of Pittsburgh Pittsburgh, PA United States; 5 Department of Informatics and Networked Systems, School of Computing and Information University of Pittsburgh Pittsburgh, PA United States; 6 Department of Biomedical Informatics, School of Medicine University of Pittsburgh Pittsburgh, PA United States

**Keywords:** intimate partner violence, text mining, social media, online health communities, linguistic features

## Abstract

**Background:**

Intimate partner violence (IPV) is an underreported public health crisis primarily affecting women associated with severe health conditions and can lead to a high rate of homicide. Owing to the COVID-19 pandemic, more women with IPV experiences visited online health communities (OHCs) to seek help because of anonymity. However, little is known regarding whether their help requests were answered and whether the information provided was delivered in an appropriate manner. To understand the help-seeking information sought and given in OHCs, extraction of postings and linguistic features could be helpful to develop automated models to improve future help-seeking experiences.

**Objective:**

The objective of this study was to examine the types and patterns (ie, communication styles) of the advice offered by OHC members and whether the information received from women matched their expressed needs in their initial postings.

**Methods:**

We examined data from Reddit using data from subreddit community r/domesticviolence posts from November 14, 2020, through November 14, 2021, during the COVID-19 pandemic. We included posts from women aged ≥18 years who self-identified or described experiencing IPV and requested advice or help in this subreddit community. Posts from nonabused women and women aged <18 years, non-English posts, good news announcements, gratitude posts without any advice seeking, and posts related to advertisements were excluded. We developed a codebook and annotated the postings in an iterative manner. Initial posts were also quantified using Linguistic Inquiry and Word Count to categorize linguistic and posting features. Postings were then classified into 2 categories (ie, matched needs and unmatched needs) according to the types of help sought and received in OHCs to capture the help-seeking result. Nonparametric statistical analysis (ie, 2-tailed *t* test or Mann-Whitney *U* test) was used to compare the linguistic and posting features between matched and unmatched needs.

**Results:**

Overall, 250 postings were included, and 200 (80%) posting response comments matched with the type of help requested in initial postings, with legal advice and IPV knowledge achieving the highest matching rate. Overall, 17 linguistic or posting features were found to be significantly different between the 2 groups (ie, matched help and unmatched help). Positive title sentiment and linguistic features in postings containing health and wellness wordings were associated with unmatched needs postings, whereas the other 14 features were associated with postings with matched needs.

**Conclusions:**

OHCs can extract the linguistic and posting features to understand the help-seeking result among women with IPV experiences. Features identified in this corpus reflected the differences found between the 2 groups. This is the first study that leveraged Linguistic Inquiry and Word Count to shed light on generating predictive features from unstructured text in OHCs, which could guide future algorithm development to detect help-seeking results within OHCs effectively.

## Introduction

Intimate partner violence (IPV) is one of the most traumatic, multifaceted, and widespread public health challenges that jeopardizes women’s health in a life span. Statistics show that 1 in 3 women have experienced some form of physical violence from their intimate partner in their lifetime [1]. Inequitably, women who have experienced IPV face more severe challenges owing to child-rearing obligations, financial dependence, and IPV-related mental health problems [2-4]. These women can be more difficult to treat compared with those experiencing other health issues, as great societal pressure to protect the peace and reputation of their families is exerted on women. Owing to shame and guilt, women refrain from asking for formal assistance because they believe that reporting IPV could put their family’s stability and privacy at risk. Therefore, the magnitude of IPV cases is consistently underreported in public data.

Successful help seeking could promote mental healing and improve the coping mechanisms, quality of life, and resilience of these women following traumatic and abusive experiences [5-8]. According to a previous study, women with IPV experience requested assistance with safety planning, financial aspects, childbearing issues, and health issues related to physical and emotional abuse [9]. Notably, they tended to seek assistance from close friends and family members rather than from institutional agencies such as health care agencies and the police [10].

Given the proliferation of computer literacy, it is convenient for those women to share their struggles and ask for guidance by using an anonymous web-based account. Online health communities (OHCs), which are web-based platforms created for a particular group of individuals dealing with a specific illness or public health issue, may help eliminate shameful and guilty feelings from in-person help-seeking processes. Tanis [11] delineated that the anonymity and deinhibiting effects of OHCs are beneficial to the self-disclosure of women with IPV experiences. According to Moors and Webber [12], OHCs provide a secure, easily accessible, and prompt response environment where these women could openly discuss sensitive family matters and traumatic experiences.

Nevertheless, not all attempts and outcomes of help seeking are desirable. As shown in previous studies, women with IPV experiences are at risk of secondary exposure to violence. For example, survivor-blaming and critical comments from friends or police officers are the main catalysts reported in recent literature [13,14]. Web-based platforms have been criticized for misinformation and selfish responses to individuals who disclose their abusive experiences. Moreover, OHC members may misuse the freedom offered by flexible OHC guidelines to make careless responses or offer unsolicited advice, including recommendations for women to leave their abusive relationships or call law enforcement agency without cautious and thorough planning, which may lead to far-reaching repercussions such as homicide [15]. As such, providing constructive and empowering responses in a nonjudgmental manner is the backbone of facilitating help-seeking initiatives among women with IPV experience.

During the COVID-19 pandemic, IPV cases surged to alarming levels as a result of the quarantine orders and strict social distancing policy [16]. López et al [17] reported the increased use of social networking sites, such as Twitter. Lyons and Brewer [18] specifically examined the contents of a particular OHC on Reddit and reported IPV survivors’ experience during the lockdown, including service disruption and preparation to leave their abusers. As a result of these unprecedented responses on OHCs, it is critical to examine whether these responses made by OHC members are helpful to women with traumatic IPV experiences. However, little is known about the types of advice provided and the manners in which OHC members offer guidance to women with IPV experience. There is also a lack of clear understanding of the number of women in OHCs who successfully received relevant responses from OHC members or what types of assistance are most frequently provided. Without bridging this knowledge gap, it will be challenging to evaluate the value of OHCs for women who worry about privacy, self-doubt, and shame in help seeking. Therefore, this study aimed to examine the types and patterns (ie, communication styles such as directive, assertive, and supportive) of the advice offered by OHC members and whether the information received by women matched their expressed needs in their initial postings. The research questions of this study included the following:

1. What types of advice do OHCs give to women with IPV experiences?
2. What patterns of communication were presented by OHC members to build the credibility of the advice (ie, how do OHC members construct their comments to convince others to take their advice)?
3. How do women with IPV experiences receive the help they sought from OHCs (ie, are there specific ways they prefer to receive help)?
4. What types of needs were mainly received and did it match with their expressed needs?

## Methods

### Study Design

This was a descriptive, exploratory study to explore the types of help received by women with IPV experiences in OHCs.

### Data Source

Data were collected from a social networking site, Reddit, to understand the types of help received by women with IPV experiences in OHCs. Reddit is a platform for knowledge and information exchange based on a wide range of topics categorized into subreddit communities, denoted as r/. This study selected r/domesticviolence as the data source to understand how women received help after sharing their IPV experience. This subreddit community was created in 2010 and had >22,000 members at the time of data collection. There are 2 rationales for choosing Reddit as our data source. First, the Reddit policy allows free-text entry without word limits in user postings. Second, the temporary “throwaway” account feature is important for women to protect their privacy after disclosure on Reddit. More comprehensive and detailed information about users’ perspectives can be extracted from postings and comments on Reddit compared with other OHCs.

### Inclusion and Exclusion Criteria

Regarding inclusion criteria, we included postings that were self-disclosed as written by adult women (ie, aged ≥18 years) with IPV experiences and sought help to solve IPV-related issues. However, postings that were written in a non-English language, posts written by underage women or women with no abuse experience, posts without help-seeking attempts, and advertisements were excluded from our data set.

### Data Structure

Data were collected between November 14, 2020, and November 14, 2021, during the COVID-19 pandemic, and the username, user number, URL, post title, post content, score (ie, the number of upvotes minus the number of downvotes in the postings), ups (ie, the number of upvotes), downs (ie, the number of downvotes), and comments were extracted using the Python program package (version 3.8; Python Software Foundation). All usernames were deidentified and replaced with random user IDs for protection of privacy.

Postings were ranked based on the number of comments and number of times the original poster (OP) returned to the thread. To achieve the objective of this study, we further excluded postings without any comments to focus on the comments’ quality and types of advice given to women with IPV experiences.

### Data Analysis

#### Data Annotation

With reference to the latest help-seeking literature published by Sivagurunathan et al [19], a codebook was created by the first author (VH) to guide the annotation process. Overall, 2 nursing researchers annotated the data set and crosschecked with 2 other undergraduate students for quality assurance. Postings with ambiguous content or unclear information were further screened by the first author (VH) and verified by IPV domain experts (RC and JCC). Once the codes for help-seeking behavior have been saturated from the data set, the first author and domain experts finalized the codebook. The annotation codebook included all the variables of interest, such as types of help received (ie, information and emotional), networking offer (ie, OHC members provide networking opportunity in their responses), and experience sharing (ie, OHC members provide their own experience in their responses).

#### Reliability of the Coded Data Set

The codebook provided clear guidelines about how each item should be annotated. The entire annotation process was performed using Excel (Microsoft Corp). All annotators were required to highlight the sentence in each posting, based on initial posts and comments, respectively. For information and emotional support, annotators were required to highlight the clues for each type of help received for quality assurance. Overall, 2 nursing annotators (ie, principal investigator and nursing undergraduate student) coded the complete data set and discussed discrepancies on a weekly or biweekly basis. Then, 2 other undergraduate annotators in psychology and nursing major with understanding of the IPV rechecked the results and screened the major discrepancies for further discussion. The final results were discussed and verified by 2 domain experts in IPV research RC and JCC. To ensure the reliability of our coded data set, Cohen κ agreement was calculated using the SPSS statistics software package [20]. The Cohen κ agreement obtained was 0.66, which indicates a substantial agreement between annotators.

#### Descriptive Statistics

The types of help (ie, information and emotional), networking offer, and experience sharing were quantified using descriptive statistics (ie, frequency and percentage).

#### Thematic Analysis

On the basis of the thematic analysis steps outlined by Brooks et al [21], the comments on posts were qualitatively analyzed to understand the themes of help-seeking behavior among women with IPV experiences. Moreover, this study followed the Reddit qualitative thematic analysis framework designed by Caplan and Purser [22]. The project leader read through the postings and responses independently to highlight the sentences about help seeking. Then, researchers were instructed to group similar help-seeking responses into different hierarchical relationships. All discrepancies were discussed, and the hierarchy was reorganized in an iterative manner. Multiple annotations and themes were coded as mutually inclusively when applicable, as multiple types of help were sometimes extracted from the same comment. The final thematic codebook and hierarchy were verified by IPV domain experts (RC and JCC).

### Matching Needs

To count the number of needs matched and unmatched in the same posting, 4 variables were created initially (ie, matched, unmatched, partly matched, and broad coverage). However, as we only annotated 3.2% (8/250) of the postings with partially matched needs, we further collapsed the variables by merging “partly matched” and “broad coverage” into “matched.” Therefore, a dichotomized variable was created, and we further explored the linguistic features of help seeking (ie, matched needs or unmatched needs). In this study, “matched needs” refer to the situations in which the OP receives the information they requested from the comments. In contrast, “unmatched needs” refer to situations in which the OP did not receive the information they requested from the comments.

### Analysis of Linguistic Features

Initial posting features include the scores, number of comments, links shared, number of words on the initial post, and title sentiment. New variables were computed, such as the number of emojis used and the title sentiment score, to enrich the linguistic features in the data set. Emojis were counted using Python, and title sentiment score was calculated using Text2Data plug-in on Excel. All initial post content was quantified and analyzed using the Linguistic Inquiry and Word Count (LIWC) tool, where 93 categories were included, ranging from emotional affect, cognitive process, self-focus, perceptions, and so on. The LIWC tool counts the frequency of words in each category and transforms qualitative text data into quantitative data to understand the psychological state and communication style of individuals through their writing. LIWC has also been applied to extract the linguistic cues for knowledge adoption, information adoption, and perceived helpfulness of web-based health reviews [23,24] and has been used for understanding the psychological behavior of people in OHCs [25].

Postings were classified into 2 groups—matched needs and unmatched needs. The linguistic features were compared between these 2 groups using parametric and nonparametric tests according to the parameter listed in LIWC. If the variables obtained fit the requirement of a parametric test, a 2-tailed *t* test was conducted. A Mann-Whitney *U* test was used for variables that violated the normality and assumptions test. All statistical analyses were conducted using IBM SPSS Statistics for Windows (version 26; IBM Corp), with a statistical significance assumed for *P* values <.05 [20]. Cohen *d* was used to quantify the effect size between the 2 groups.

### Ethical Considerations

As web-based data were publicly available for this study, an approval for exemption was obtained from the institutional review board at the University of Pittsburgh (MOD20030179-002).

## Results

### Overview

**Figure 1:**
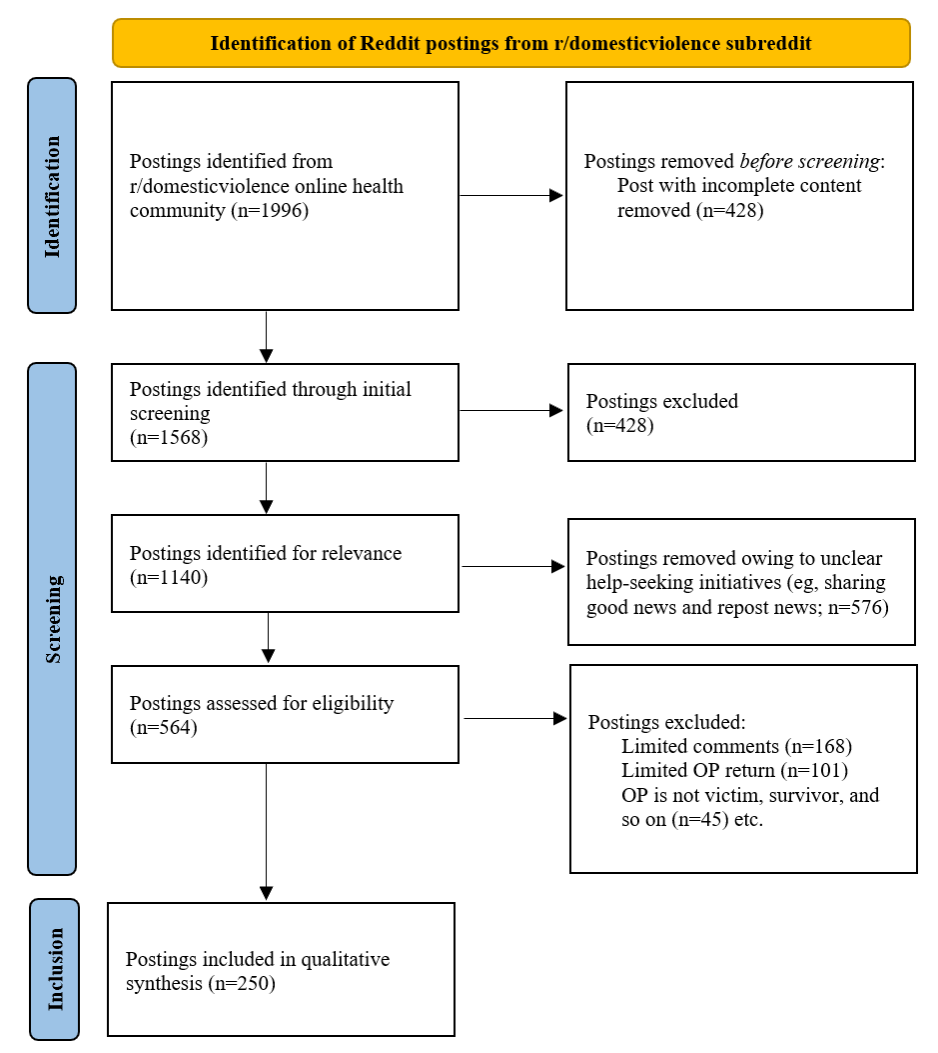
PRISMA (Preferred Reporting Items for Systematic Reviews and Meta-Analyses) flowchart of Reddit post screening among women with intimate partner violence experience between 2020 and 2021. OP: original poster.

A total of 1996 postings were collected from Reddit. In the initial screening of the 1996 postings, 1568 (78.56%) postings were selected and 1140 (57.11%) postings were identified for relevance. After excluding the postings without comments or OP return, 21.93% (250/1140) of the postings were annotated quantitatively and analyzed qualitatively using thematic analysis (Figure 1).

### Types of Help-Seeking Advice Received

The types of help received were divided into information support and emotional support. Overall, 97.2% (243/250) of the postings received information support, whereas 87.6% (219/250) received emotional support (Table 1). Among the information support received, the top 5 common types of information help received were IPV knowledge (414/1568, 26.4%), IPV shelter (242/1568, 15.43%), legal information (190/1568, 12.12%), health care information (187/1568, 11.93%), and safety planning (131/1568, 8.35%). In terms of emotional support, the top 3 were encouragement (570/1173, 48.6%), empathy (174/1173, 14.83%), and mutual understanding (159/1173, 13.55%). Approximately one-third (74/250, 29.6%) of the comments received networking offer, and more than two-thirds (195/250, 78%) of the comments received experience sharing from OHC members.

**Table 1:** Types of help received by women with intimate partner violence (IPV) experiences in online health communities from 2020 to 2021.

		Posts, n (%)
**Information support received (n=250)**		
	Yes	243 (97.2)
	No	7 (2.8)
**Types of information help received (multiple responses were allowed; n=1568)**		
	Shelter, IPV center, or agency	242 (15.4)
	Legal	190 (12.1)
	Childbearing	59 (3.8)
	Police	110 (7)
	Wound assessment, or record	7 (0.5)
	IPV report procedure, or documentation	77 (4.9)
	Safety planning	131 (8.4)
	Finance	25 (1.6)
	Housing	37 (2.4)
	Health care information	187 (11.9)
	IPV survivors’ network, or online support groups	32 (2)
	IPV knowledge	414 (26.4)
	Communication	17 (1.1)
	Miscellaneous	40 (2.6)
**Emotional support received (n=250)**		
	Yes	219 (87.6)
	No	31 (12.4)
**Types of emotional help received (multiple responses were allowed; n=1173)**		
	Love	77 (6.6)
	Empathy	174 (14.8)
	Mutual understanding	159 (13.6)
	Reassurance	114 (9.7)
	Acceptance	79 (6.7)
	Encouragement	570 (48.6)
**Experience sharing (n=250)**		
	Yes	195 (78)
	No	55 (22)
**Networking offer (n=250)**		
	Yes	74 (29.6)
	No	176 (70.4)

### Themes of Help Received

Among the 250 postings, a total of 7 themes and 15 subthemes were identified from the comments. All authors reviewed and confirmed the hierarchy of each theme and subtheme. Overall, seven main themes were generated, including (1) experience sharing, (2) emotional empowerment, (3) IPV knowledge display, (4) advice type, (5) clarification of scenario, (6) networking offer, and (7) daily self-care tips. The 15 subthemes that were found are shown in Multimedia Appendix 1.

### Types of Help Matched

Overall, 80% (200/250) of the postings received matching help from OHCs (Table 2). Among all types of help annotated as matching help, only wound documentation was excluded from the list because no women sought wound information in our data sets, but some comments include wound information when they provide advice regarding safety planning instead. Table 3 shows that the highest 2 information needs matched were legal (90%) and IPV knowledge (86%). In terms of emotional support, 92.5% of the postings were successfully matched with emotional help. However, finance (57%), communication (53%), housing (50%), health care information (48%), and IPV survivors’ network (33%) received the least information help among postings with matched needs.

**Table 2:** Matching needs annotated among women with intimate partner violence in online health communities (n=250).

Categories			Posts, n (%)
Matched			146 (58.4)
Unmatched			50 (20)
Partly matched			8 (3.2)
Broad coverage			46 (18.4)
**After merging categories**			
	Matched	200 (80)	
	Unmatched	50 (20)	

**Table 3:** Information support matched rate among women with intimate partner violence (IPV) in online health communities (n=250).

Types of support	Matched rate, %
IPV shelters and agency	83
Legal	90
Childbearing	66
Police	80
IPV report procedure	75
Safety planning	78
Finance	57
Housing	50
Health care	48
IPV survivors network	33
IPV knowledge	86
Communication	52
Miscellaneous	82

### Linguistic Features

We used the LIWC software to examine the use of 93 prespecified dictionaries (lists of words) between the 2 groups (ie, matched help and unmatched help). Multimedia Appendix 2 displays the descriptive statistics of posting features from the annotated data and linguistic features from the LIWC tools among the 250 postings. All significant linguistic features between the 2 groups are displayed in Table 4. The average length of postings was 298 (SD 257) words, and no postings were generated from the same user ID (ie, person). Among the 250 initial postings, a total of 18 features (n=3, 17% posting features and n=15, 83% linguistic features) were found with statistically significant differences between the 2 groups. Postings with negative sentiment, more comments, and more number of words in comments were easy to receive matched help in OHCs. Postings containing words related to health (eg, medic, patients, physician, and health) or wellness (eg, healthy, gym, exercise, and diet) were found to be more difficult to receive matched help in OHCs.

**Table 4:** Statistically significant variables of Linguistic Inquiry and Word Count categories with help-seeking results (ie, matched needs vs unmatched needs) among women with intimate partner violence experience in online health communities (n=250) from 2020 to 2021.

Features			Matched needs, mean (SD)		Unmatched needs, mean (SD)		Mean difference						
							Mann-Whitney U test (*df*)		*t* test (*df*)		*P* value		Cohen *d*
**Posting features**													
	Number of comments	*14.08*^a^ (8.45)		10.14 (2.98)		N/A^b^		3.243 (248)		<.001		0.513	
	Number of words in comments	*1732.01* (1146.03)		1463.84 (803.16)		N/A		1.561 (248)		.05		0.247	
	Title sentiment^c^	−0.05 (0.41)		*0.07* (0.32)		1.561 (248)		N/A		.05		−0.315	
**Linguistic features**													
	Culture (eg, car, united states, govern, and phone)	*0.31* (0.65)		0.12 (0.23)		2.074 (248)		N/A		<.001		0.328	
	Political (eg, legal, court, law, and congress)	*0.02* (0.08)		0 (0)		1.510 (248)		N/A		.003		0.239	
	Technology (eg, Wi-Fi, computer, and phone)	*0.29* (0.64)		0.11 (0.23)		1.948 (248)		N/A		.001		0.308	
	Politeness (eg, thank, please, and thanks)	*0.40* (1.62)		0.16 (0.39)		1.037 (248)		N/A		.049		0.164	
	Health (eg, medic, patients, physician, and health)	0.87 (0.98)		*1.26* (1.70)		−2.137 (248)		N/A		.02		−0.338	
	Wellness (eg, healthy, gym, exercise, and diet)	0.04 (0.14)		*0.21* (0.67)		−3.376 (248)		N/A		.047		−0.534	
	Emotional anxiety (eg, worry, fear, afraid, and nervous)	*0.42* (0.68)		0.26 (0.53)		1.551 (248)		N/A		.04		0.245	
	Lifestyle (eg, work, home, school, and working)	*1.85* (1.69)		1.29 (1.24)		2.223 (248)		N/A		.03		0.351	
	Leisure (eg, TV, cook, chat, fun, and play)	*0.17* (0.37)		0.09 (0.22)		1.435 (248)		N/A		.048		0.227	
	Home (eg, home, lawn, room, and furniture)	*0.46* (0.66)		0.25 (0.41)		2.132 (248)		N/A		.006		0.337	
	Work (eg, work, school, working, and class)	*0.87* (1.12)		0.52 (0.63)		2.112 (248)		N/A		.004		0.334	
	Conversation (eg, yeah, oh, yes, and okay)	*0.55* (0.92)		0.22 (0.43)		2.444 (248)		N/A		<.001		0.386	
	Netspeak (eg, I know, u, lol, and haha)	*0.42* (0.85)		0.19 (0.39)		0.010 (248)		N/A		.005		0.032	
	Assent (eg, yeah, yes, okay, and ok)	*0.12* (0.41)		0.03 (0.08)		1.577 (248)		N/A		.004		0.249	

^a^Italicized text represents higher tendency on associated features.

^b^N/A: not applicable.

^c^Title sentiment was assessed on a scale ranging from −1 to 1, with negative values indicating negative sentiment and vice versa.

## Discussion

### Principal Findings

This is the first study to investigate the types of help and advice presented in OHCs among women with IPV experience through qualitative analysis and quantitative linguistic analysis in a western context. We found that most women received information and support that matched the type of help they requested in their original postings. In addition, the majority of the postings (178/250, 71.2%) received response in which OHC members shared their own experiences, mistakes, and lessons learned.

The willingness of OHC members to share their own IPV experiences in response to posters’ requests for help correlates with one of the presumed reasons for the existence of this subreddit community—to serve as a platform for experience exchange and support for people with IPV experience or other passion for the topic. By sharing their own experiences, OHC members illustrate credibility to offer advice to the OP. Our findings aligned with a previous study by Yan and Tan [26] regarding OHC members’ perceptions and experiences about the medical treatment for mental health were related to the perceived effectiveness of treatment among other OHC members. Similarly, Fan et al [27] pinpointed that the experience-sharing behavior was an approach to building trust with other OHC members. In the IPV context, Krisvianti and Triastuti [28] concluded that the experience-sharing behavior facilitates social support and empowerment among women, whereas Afdal et al [29] reported that using OHC can improve life satisfaction through experience sharing.

In addition, emotional empowerment was another important theme identified in the OHC comments. Our study found that OHC members provided different forms of emotional empowerment, such as encouragement, empathy, and reassurance when the OP was lost and helpless, as expressed in the initial postings. A previous study pinpointed a strong association between emotional support and IPV severity among women [30]. Female survivors who either experienced physical abuse or had unwanted sex with their partner have 2.28 higher odds of receiving less emotional support, indicating a great demand of emotional needs in this vulnerable population. Lyons and Brewer [18] also reported the themes related to the IPV survivors’ experience during the COVID-19 pandemic [17]. Specifically, one of their themes highlighted service disruption such as IPV shelters, counseling service, and emergency room in hospital, which could potentially undermine the help-seeking initiatives from women. Therefore, it is possible that women with IPV experience shifted their help-seeking attempts to OHC web-based environment, where OHC members could provide emotional empowerment with a timely response regardless of geographical and time restrictions. Moreover, we found that women with IPV experience used to seek help by asking OHC members to prove whether they were acting normal or not overreacting to what happened in an abusive relationship. Our findings were consistent with other OHCs’ findings in different contexts ranging from ovarian cancer [31], breast cancer [32], and pregnancy loss [33]. In the male IPV context, Sivagurunathan et al [19] also reported the importance of emotional validation in help seeking after sexual assault. Therefore, our study consolidated that emotional empowerment and validation are essential for OHC help seeking among women with IPV experience.

Furthermore, it is noteworthy that demonstrating IPV knowledge in the comments is a strategic way to present advice with credibility in OHCs among women. Our findings demonstrated that OHC members used to cite IPV statistics from well-known organizations and highlight their IPV knowledge (eg, trauma bonding, stages of abuse, reactive abuse, and warning signs of IPV such as strangulation and choking) to build advice credibility. When women were confused about their current IPV risks and condition, OHC members presented their IPV knowledge to guide them regarding how to recognize warning signs and navigate them to related resources.

Interestingly, our analysis identified 2 advice styles—directive and emphasizing empowerment and advice form. Directive styles were commonly found to urge OP to make decisions and seriously listen to OHC members in postings with multiple alerts labeled by Reddit, such as multiple triggers, physical abuse, and suicidal risk. Some OHC members reacted emotionally without consideration for OP’s feelings as they were not well trained with trauma-informed care strategies. Given the disinhibition effect on OHCs [34], OHC members may not take responsibility for what they suggested to OP owing to the anonymity and invisibility environment in OHC.

Regarding comments identified as having more empowerment and advice given by OHC members, this advice style is viewed as more appropriate, considerate, safe, and helpful for this vulnerable population. This result aligned with that of a previous study that illustrated the importance of emotional support for women with IPV experience [30]. Nevertheless, a previous study reported that 37% of the comments contained harmful messages with survivor-blaming intentions in OHCs [15]. As we prioritized postings with the highest number of comments, we filtered postings without comments or those without OP returning to initial postings. Therefore, it is possible that some negative responses were not well captured in our analysis.

Moreover, we adopted the LIWC tool to quantify our annotation results and compare the help-seeking results from a computational point of view at the word level. As OHC members may offer help primarily based on the textual description in a web-based environment, traditional, in-person, help-seeking factors such as facial expressions and differences in tone of voice may not apply. Therefore, our results shed more light on the linguistic and posting features that could affect help-seeking results in OHCs. As such, displaying politeness in the initial postings may indicate that OHC members will offer help more comfortably. In contrast, our results found that postings with wordings associated with health and wellness have low chance of successful help seeking in OHCs. One of the possible explanations is that OHC members are not well trained in medical terminologies and do not have the related expertise to address postings with complicated medical terminologies. According to the scoping review conducted by Perry et al [35], only 1 OHC featured a moderator with health care professional qualifications in the related field, whereas most OHCs were moderated by volunteers or survivors of IPV. Hence, postings with health or wellness wordings may not be quickly addressed by OHC members. Future studies can consolidate these results by inviting OHC moderators for an interview to understand how they offer help based on textual descriptions.

We also found that posting titles with negative sentiment were more likely to receive help than using wordings with positive sentiment. Given the anonymity provided, women may feel more comfortable in seeking help urgently in OHCs to receive real-time responses from OHC members [36]. Liu et al [37] reported that patients with psychological diseases posted more negative words and emotions than others. More importantly, 49.5% of women were shown to develop psychological illnesses after experiencing IPV [38]. It is possible that those who post negative sentiments may have more complex information and emotional needs than others, where OHC members may address their needs and answer their questions easily. Therefore, title sentiments should be considered as the primary indicator of IPV severity and urgency in a web-based setting such as OHC.

### Implications

This study has the potential to contribute to the management of women with IPV experience from a clinical perspective. The results obtained from this study demonstrated the usefulness of OHCs in providing access to IPV knowledge and a platform for experience exchange for women; therefore, clinicians are encouraged to assess their perceived IPV knowledge (ie, potential risks and consequences of IPV, potential danger, and safety strategies to protect themselves) before referral to other departments or planning for next appointment. Clinicians may provide well-known resources that are available in the OHC and appropriate to their personal situation. However, given that professionals do not filter the comments and suggestions from OHCs, clinicians should remind women to avoid taking all the advice personally after seeking help in OHCs. Reading other OHC members’ abusive experiences could retraumatize women with previous IPV experience. If some comments are typed in a directive manner with harsh wordings, clinicians could advise women to set a time-out period from reading these comments. Moreover, OHCs, when moderated by IPV experts, have the capability to identify comments with a reproachful tone and provide tailored resource navigation based on the specific geographical location of users.

In addition, IPV agencies and emergency departments in hospitals should also establish a protocol to enable nurses, medical social workers, and other health care professionals to identify when they should refer women with IPV experience to OHC. For example, health care professionals can streamline the process by providing OHC information to at-risk women after referring them to in-person support groups or IPV-related agencies. Moreover, women should be advised about how to seek help in OHCs effectively by emphasizing the value of choosing a post title with negative connotations and seeking help politely in the initial postings. Furthermore, a recent review discussed how an automated system can assist IPV detection [39]. Our study is a starting point for developing future IPV automatic system interventions that allow users to search for comparable experiences effectively. For example, IPV experience can be further categorized based on positive and negative feedback by domain experts and a computational algorithm can be trained to classify relatable experiences based on the needs of women, which could facilitate a better help-seeking experience in OHCs.

### Limitations

It is noteworthy that the results of this study should be interpreted with caution. First, owing to Reddit’s privacy policy, we could not collect the users’ demographic data, such as race, age, and educational background. Second, Reddit is only widely recognized in a few English-speaking nations, such as the United States, Canada, the United Kingdom, and Australia. Therefore, conclusions from this study are not fully generalizable to women with IPV experiences from other regions. Third, this study examined the type and pattern of advice provided by OHCs in 1 subreddit community. Future research endeavors should include more OHCs for analysis and comparisons. In addition, this study depended on the details provided in users’ initial posts during the screening procedure to determine whether the OP was female and aged >18 years. However, some users may have hidden their actual sex and age because of privacy concerns. Moreover, our study reranked the postings based on their number of comments and the number of times the OP returned to the initial postings. As such, it is highly possible that some negative comments have not been analyzed thoroughly in our data set. Future studies should evaluate the risky suggestions or harmful comments from postings and analyze the potential danger score with an IPV domain expert to prevent retraumatization in these vulnerable populations. An additional constraint pertains to the data collection period—from November 2020 to November 2021. During this period, the COVID-19 pandemic posed significant challenges, such as quarantine orders, business closures, and limited access to health care services. As the World Health Organization has since declared that COVID-19 is no longer a global emergency, it is plausible that help-seeking behaviors and available resources may have evolved after the time of data collection.

### Conclusions

This study elicits empirical data about the types of help given to women with IPV experience and what constructs the advice credibility among OHC members. Our findings show that OHC members often describe their own relevant experiences before highlighting the IPV knowledge to the OPs. In this manner, OHCs offer an accessible platform for knowledge acquisition and experience sharing for women who feel baffled and distressed following their IPV experience. Our study has implications for developing digital textual initiatives to educate women about IPV and exploring computation methods to connect women with others who have similar experiences. In addition, we assessed linguistic variations between successful and unsuccessful help-seeking postings in the OHC. Our findings demonstrate how linguistic variations in politeness and title sentiment may affect to what extent OHC members meet the help seekers’ needs. Future studies should examine the language variations between postings with the most and the fewest comments. Overall, the responses from OHCs are constructive to provide various sources of help for women with IPV experience, including emotional validation, IPV knowledge, and relatable experiences, so that they can identify the warning signs and potentially far-reaching consequences of their IPV experiences.

## Appendix

Multimedia Appendix 1 Thematic analysis to explore the help received by women with intimate partner violence experiences in online health communities from 2020 to 2021.

Multimedia Appendix 2 Descriptive statistics of posting features from annotated data and linguistic features from Linguistic Inquiry and Word Count.

## Abbreviations

IPV: intimate partner violence
LIWC: Linguistic Inquiry and Word Count
OHC: online health community
OP: original poster

## Data Availability

The data sets generated and analyzed during this study are available from the corresponding author upon reasonable request.

## References

[1] Smith SG, Zhang X, Basile KC, Merrick MT, Wang J, Kresnow MJ, Chen J (2018). The national intimate partner and sexual violence survey: 2015 data brief: updated release. National Center for Injury Prevention and Control Centers for Disease Control and Prevention.

[2] Scheffer Lindgren MS, Renck B (2009). Intimate partner violence and the leaving process: Interviews with abused women. Int J Qual Stud Health Well-being.

[3] Johnson DM, Zlotnick C (2009). HOPE for battered women with PTSD in domestic violence shelters. Prof Psychol Res Pr.

[4] Jones L, Hughes M, Unterstaller U (2016). Post-Traumatic Stress Disorder (PTSD) in victims of domestic violence: a review of the research. Trauma Violence Abuse.

[5] Bennett L, Riger S, Schewe P, Howard A, Wasco S (2004). Effectiveness of hotline, advocacy, counseling, and shelter services for victims of domestic violence: a statewide evaluation. J Interpers Violence.

[6] Frías SM (2013). Strategies and help-seeking behavior among Mexican women experiencing partner violence. Violence Against Women.

[7] Goodkind JR, Gillum TL, Bybee DI, Sullivan CM (2003). The impact of family and friends’ reactions on the well-being of women with abusive partners. Violence Against Women.

[8] Orchowski LM, Gidycz CA (2015). Psychological consequences associated with positive and negative responses to disclosure of sexual assault among college women: a prospective study. Violence Against Women.

[9] Satyen L, Piedra S, Ranganathan A, Golluccio N (2018). Intimate partner violence and help-seeking behavior among migrant women in Australia. J Fam Violence.

[10] Parvin K, Sultana N, Naved RT (2016). Disclosure and help seeking behavior of women exposed to physical spousal violence in Dhaka slums. BMC Public Health.

[11] Tanis M (2008). Health-related on-line forums: what's the big attraction?. J Health Commun.

[12] Moors R, Webber R (2023). The dance of disclosure: online self-disclosure of sexual assault. Qual Soc Work.

[13] Nagy V (2017). Narrative construction of sexual violence and rape online. Int J Crime Justice Soc Democr.

[14] Powell A (2015). Seeking rape justice: formal and informal responses to sexual violence through technosocial counter-publics. Theor Criminol.

[15] Whiting J, Dansby Olufowote RD, Cravens-Pickens JD, Banford Witting AB (2019). Online blaming and intimate partner violence: a content analysis of social media comments. Qual Rep.

[16] Evans ML, Lindauer M, Farrell ME (2020). A pandemic within a pandemic - intimate partner violence during COVID-19. N Engl J Med.

[17] López G, Bogen KW, Meza-Lopez RJ, Nugent NR, Orchowski LM (2022). #DomesticViolence during the COVID-19 global pandemic: an analysis of public commentary via Twitter. Digit Health.

[18] Lyons M, Brewer G (2022). Experiences of intimate partner violence during lockdown and the COVID-19 pandemic. J Fam Violence.

[19] Sivagurunathan M, Walton DM, Packham T, Booth R, MacDermid J (2021). "Punched in the balls": male intimate partner violence disclosures and replies on reddit. Am J Mens Health.

[20] (2017). IBM SPSS statistics for Windows, version 26.0. IBM Corp.

[21] Brooks J, McCluskey S, Turley E, King N (2015). The utility of template analysis in qualitative psychology research. Qual Res Psychol.

[22] Caplan MA, Purser G (2019). Qualitative inquiry using social media: a field-tested example. Qual Soc Work.

[23] Chen L (2020). The impact of linguistic cues on knowledge adoption in online knowledge communities: a signaling theory perspective. Proceedings of the 26th Americas Conference on Information Systems.

[24] Shah AM, Ali M, Qayyum A, Begum A, Han H, Ariza-Montes A, Araya-Castillo L (2021). Exploring the impact of linguistic signals transmission on patients' health consultation choice: web mining of online reviews. Int J Environ Res Public Health.

[25] McDonnell M, Owen JE, Bantum EO (2020). Identification of emotional expression with cancer survivors: validation of linguistic inquiry and word count. JMIR Form Res.

[26] Yan L, Tan Y (2017). The consensus effect in online health-care communities. J Manag Inf Syst.

[27] Fan H, Smith SP, Lederman R, Chang S (2010). Why people trust in online health communities: an integrated approach. Proceedings of the 21st Australasian Conference on Information Systems.

[28] Krisvianti S, Triastuti E (2020). Facebook group types and posts: Indonesian women free themselves from domestic violence. J Media Commun Res.

[29] Afdal A, Arnaldi A, Nirwana H, Alizamar A, Zikra Z, Ilyas A, Fikri M (2019). Increasing life satisfaction of domestic violence victims through the role of supporting group therapy on social media. Adv Soc Sci Educ Humanit Res.

[30] Hui V, Constantino RE (2021). The association between life satisfaction, emotional support, and perceived health among women who experienced intimate partner violence (IPV) - 2007 behavioral risk factor surveillance system. BMC Public Health.

[31] Benson JJ, Oliver DP, Washington KT, Rolbiecki AJ, Lombardo CB, Garza JE, Demiris G (2020). Online social support groups for informal caregivers of hospice patients with cancer. Eur J Oncol Nurs.

[32] Yoo W, Namkoong K, Choi M, Shah DV, Tsang S, Hong Y, Aguilar M, Gustafson DH (2014). Giving and receiving emotional support online: communication competence as a moderator of psychosocial benefits for women with breast cancer. Comput Human Behav.

[33] Andalibi N, Garcia P (2021). Sensemaking and coping after pregnancy loss: the seeking and disruption of emotional validation online. Proc ACM Hum Comput Interact.

[34] Suler J (2004). The online disinhibition effect. Cyberpsychol Behav.

[35] Perry A, Pyle D, Lamont-Mills A, du Plessis C, du Preez J (2021). Suicidal behaviours and moderator support in online health communities: a scoping review. BMJ Open.

[36] Chandan JS, Thomas T, Bradbury-Jones C, Russell R, Bandyopadhyay S, Nirantharakumar K, Taylor J (2020). Female survivors of intimate partner violence and risk of depression, anxiety and serious mental illness. Br J Psychiatry.

[37] Liu M, Xue J, Zhao N, Wang X, Jiao D, Zhu T (2021). Using social media to explore the consequences of domestic violence on mental health. J Interpers Violence.

[38] Blasco-Ros C, Sánchez-Lorente S, Martinez M (2010). Recovery from depressive symptoms, state anxiety and post-traumatic stress disorder in women exposed to physical and psychological, but not to psychological intimate partner violence alone: a longitudinal study. BMC Psychiatry.

[39] Hui V, Constantino RE, Lee YJ (2023). Harnessing machine learning in tackling domestic violence-an integrative review. Int J Environ Res Public Health.

