# Addendum: Examining the Supports and Advice That Women With Intimate Partner Violence Experience Received in Online Health Communities: Text Mining Approach

**DOI:** 10.2196/85800

**Published:** 2025-12-02

**Authors:** Vivian Hui, Malavika Eby, Rose Eva Constantino, Heeyoung Lee, Jamie Zelazny, Judy C Chang, Daqing He, Young Ji Lee

**Affiliations:** 1 Center for Smart Health, School of Nursing The Hong Kong Polytechnic University Hong Kong China (Hong Kong); 2 Health and Community Systems, School of Nursing University of Pittsburgh Pittsburgh, PA United States; 3 Department of Psychology Swarthmore College Swarthmore, PA United States; 4 Department of Obstetrics, Gynecology & Reproductive Sciences, and Internal Medicine University of Pittsburgh Pittsburgh, PA United States; 5 Department of Informatics and Networked Systems School of Computing and Information University of Pittsburgh Pittsburgh, PA United States; 6 Department of Biomedical Informatics, School of Medicine University of Pittsburgh Pittsburgh, PA United States

In “Examining the Supports and Advice That Women With Intimate Partner Violence Experience Received in Online Health Communities: Text Mining Approach”  the authors made one clarification.

In the originally submitted manuscript, the Ethics Approval section appeared as follows: 

*As web-based data were publicly available for this study, an approval for exemption was obtained from the institutional review board at the University of Pittsburgh (MOD20030179-002).*

This has been updated as follows: 

*As web-based data were publicly available for this study, an approval for exemption was obtained from the institutional review board at the University of Pittsburgh (MOD20030179-002).* *IRB approval was obtained prior to the subreddit’s ‘no research’ use rule, which has since been implemented*

The correction will appear in the online version of the paper on the JMIR Publications website, together with the publication of this correction notice. Because this was made after submission to PubMed, PubMed Central, and other full-text repositories, the corrected article has also been resubmitted to those repositories.

