# Supplementary material for: Examining the Supports and Advice That Women With Intimate Partner Violence Experience Received in Online Health Communities: Text Mining Approach
Source: J Med Internet Res. 2023 Oct 9;25:e48607. doi: 10.2196/48607 (PMC10594147; doi:10.2196/48607)
Supplement: Multimedia Appendix 1 [file jmir_v25i1e48607_app1.docx]

**Multimedia Appendix 1.** Thematic analysis to explore the help received by women with intimate partner violence (IPV) experiences in online health communities from 2020 to 2021.

| Themes and subthemes | | Examples extracted from postings |
| --- | --- | --- |
| **Experience sharing** | | |
|  | IPV experience | - “If I may...the longer you stay, the more danger you are in. There were a few times when my mom was very close to being killed. It took many years of abuse to reach that level of violence but each occasion only escalates to a more dangerous level.” |
|  | IPV agencies and shelters experience | - “I volunteer for an agency that has a shelter, but most of our clients do not live in the shelter. We have staff therapists, a support group, a legal advocate, and victim advocates. Perhaps these folks you have been talking to can steer you toward help that doesn’t involve staying in the shelter.” - “Domestic violence hotline or support center will never tell you what you have to do. Instead, they get you a safety plan, they tell you what resources they have. They may even help you with groceries or any services they offer to relieve the financial burden caused by the abuse. As everyone else says, it seems difficult but you do have some options. The problem is that you are probably going to have to be relentless in your efforts to get to them.” |
|  | Emotional torture from legal/shelters | - “Shelters don’t accommodate many disabilities. There are a plethora of issues with shelters, but they won’t kill you. Please work with DV services to make a safety plan.” |
|  | Ineffective counseling therapy | - “If you suspect at all there is abuse here [and you are posting in a DV sub, after all] it is never recommended to go to couples counseling with an abuser. Not only is it pointless, couples counseling generally focuses on communication. Abuse is not a communication problem. Many couples therapists are not well trained even in spotting abusers let alone treating victims. Therapists are not all the same. And abusers will often manipulate counselors into essentially helping them target you as the problem. Even if that were the case, abusers do not go to couples counseling to resolve the issue. They go to appease you to keep you in place to abuse, to listen to your feelings about when you were hurt by them, about what you want, etc all to be more effective in abusing you. They learn and misappropriate the language to use that against you as well. What they never do is go to a counselor and suddenly have a realization that they are abusive and make a complete change in their personality and behavior and even their way of thinking.” |
|  | Experience with police | - “Calling the police is Russian roulette, it depends on who deals with it. You could get a survivor of domestic abuse who does everything that they can, it could be someone who just doesn’t get it or doesn’t care. It also depends on how busy they are at the time if the victim feels comfortable opening up fully and if there is enough evidence in your case. What area of the UK are you in? Different areas have different policies and risk assessments for example some use DASH. Google your areas and do it honestly and see what you come out as. Don’t hold back, be honest. With a high-risk assessment you’re entitled to more support from the council, social services, fire service, and police but IME sometimes you need to force the risk assessment on them and bang a lot of doors to get help.” |
| **Emotional empowerment** | | |
|  | Reassurance | - “He was being aggressive, threatening, and intimidating. Even though he took his frustration out on inanimate objects you felt unsafe around him. Your decision to leave based on this is perfectly justified. You need no further reasons than that to know you have done the right thing for your safety and future happiness.” |
|  | Empathy | - “I am sorry that happened to you” - “Please know that you are not alone. Some of us know the struggle, all too well.” |
|  | Positive vibes and faith | - “I’m confident in you and your choices, that you can do what is best and future-you will look back with thanks for your decisions and actions, even the hardest ones.” |
|  | Validation | - “You have the right to feel safe. You have a right to feel however you feel.” - “You are not an asshole. You are a human being that has not been treated like a human being. It sounds like you have conflicting emotions. That is perfectly normal. Don’t beat yourself up because of conflicting emotions.” |
| **IPV knowledge display** | | |
|  | IPV terminology and types | - “Gaslighting is a strategy that can leave you deeply uncertain about your memory and judgment. That confusion and doubt is then what abusers take advantage of: they can then totally shape a survivor’s reality.” - “When an abuser tries to strangle someone in a domestic assault, it is a leading indicator that he will escalate his attacks and eventually kill his victim, says Gael Strack, a former prosecutor and founder of the Family Justice Center Alliance, which helps abuse victims.” - “Please do not discount abuse just because it is not physical. Look up emotional and mental abuse too, it’s extremely damaging, but often starts so subtle it will take you years to see it.” |
|  | IPV statistics/evidence-based knowledge | - “In the United States, victims who have been strangled are 750% more likely than other victims to be killed. Unfortunately, leaving is the most dangerous part.” - “Statistically leaving is the most dangerous time and now he knows it’s over and that she’s leaving. I don’t see him giving in so easily. He has threatened to have her killed if she leaves. I’m super concerned for the OP’s welfare.” - “A 2008 study in the Journal of Emergency Medicine found that 43% of women who were murdered in domestic assaults and 45% who were victims of attempted murder had been choked in the past year by their male partners.” |
| **Advice type** | | |
|  | Directive style | - “GET OUT NOW! Leave, and don’t look back!” - “You have to leave now! He will kill you.” - “Please call 911 and report his ass, not only did he physically assault you, but he also raped you. This isn’t worth going through like some of the other comments, forget the car, forget the money, forget physically possessions, your life is in danger! Call your sister and explain to her the situation, maybe you two can stay at another family member’s house or motel meanwhile. Contact the police and get a restraining order. I hope so much you can get away...no one deserves to go through this hell.” - “If you go back, he will eventually kill you. I’m convinced of this. These types of people prey on us because we have no self-esteem. And once they have us, they rip shreds through whatever we have to make sure we don’t leave and stop ya ding over cash, doing their Lau dry, etc.” |
|  | Emphasizing the empowerment and advice | - “I read in one of your comments that you have a family the next state over – if you can, start to make a plan to leave for there. If you have access to computers at work, google DV organizations there. Make the calls from there. If you have to use your phone, perhaps consider clearing the “past hour” of history if you’ve had to do any leaving-related searches. Hide all your important documents.” - “I understand what you’ve been through, please take good care of yourself first and consider the seeking help from close friends or your sisters. Record all the wound condition with your phones and share the location with friends to protect yourself. Trust yourself, you can do it.” |
| **Clarification of the scenario** | | |
|  | Identification of safety-related warning signs | - “The level of escalation is a WARNING SIGN shouting to strangulation means flee ASAP. On the lethally test this rates 120/140 points! It’s time to go~>~>~> usually this means your parenteral will murder you in the next 3-6 months.” - “This is a red flag. Do not ignore it. Learn from others. Be safe.” |
|  | Self-blame related | - “His reaction to your disagreement is completely out of line. It’s not your fault. At all.” - “You didn’t cause the argument.   You’re allowed to have different views from him.  You didn’t cause him to look up disturbing images.  He chose to react inappropriately to a minor disagreement.  He chose to send you threatening messages and a disturbing, sick image.”   - “The first thing to remind yourself is it is 100%, not your fault. Your abuser made their shitty choice and the burden is theirs to bear. So you didn’t make or let yourself go through anything. Forgive yourself for being so close to the situation you didn’t see it bc it didn’t happen overnight. It was gradual.” |
| **Networking offer (by OHC^a^ members to OP^b^)** | | |
|  | N/A^c^ | - “Please pm me if you want someone to talk to.” - “I am trying to address two things here...one is to offer a couple of potential solutions for you (they may or may not be helpful to you and from what you’ve written, it is clear there is much more that needs to be addressed, but there is only so much I can reply here right now), the other is to assure you that your response and feelings are fine. There is nothing wrong with you for feeling the way you do.” |
| **Daily self-care tips** | | |
|  | N/A | - “Take time to pamper yourself, color your hair, gym, sell unused, unwanted stuff on eBay, if you’re crafty make & sell stuff on Etsy, walk dogs, do shopping for elderly neighbors, put money in your pocket, & can break the mundanity of your days, change your mindset, do not put weight in his words when he’s being, particularly arseholey -if it’s safe to wait till he’s finished & ask if he’s feeling better now.” - “Upskill & do online courses, start meditation, listen to podcasts, if he yells at you or tells you who or what you are, ask why if he could do better he hasn’t? do not get in a car with him unless it’s absolutely necessary & make sure you’re visible, if you’re arty or crafty make & sell things on Etsy & lots of people find knitting, and crocheting calming. Blog everything online with pics, join a church support group & a support group for people with your health conditions.” |

^a^OHC: online health community.

^b^OP: original poster.

^c^N/A: not applicable.
