# Supplementary material for: Examining the Supports and Advice That Women With Intimate Partner Violence Experience Received in Online Health Communities: Text Mining Approach
Source: J Med Internet Res. 2023 Oct 9;25:e48607. doi: 10.2196/48607 (PMC10594147; doi:10.2196/48607)
Supplement: Multimedia Appendix 2 [file jmir_v25i1e48607_app2.docx]

***Appendix A. Descriptive statistics of postings features from annotated data
and linguistic features from LIWC.***

| Linguistic and posting features | N | | Mean | Std. Deviation |
| --- | --- | --- | --- | --- |
|  | Valid | Missing |  |  |
| SCORE | 250 | 0 | 13.44 | 12.27 |
| Number of comments | 250 | 0 | 13.29 | 7.83 |
| OP_BACK | 250 | 0 | 4.68 | 3.73 |
| COMMENTS_WORDS | 250 | 0 | 1678.42 | 1090.03 |
| TITLE_SENTI | 250 | 0 | -0.02 | 0.40 |
| LINKS_SHARED | 250 | 0 | 0.54 | 1.31 |
| EMOJI_USE | 250 | 0 | 1.26 | 2.52 |
| Word count | 250 | 0 | 298.16 | 258.17 |
| Analytic | 250 | 0 | 13.93 | 12.95 |
| Clout | 250 | 0 | 22.32 | 25.51 |
| Authentic | 250 | 0 | 70.84 | 28.67 |
| Tone | 250 | 0 | 13.34 | 18.81 |
| WPS | 250 | 0 | 19.71 | 20.71 |
| BigWords | 250 | 0 | 13.73 | 3.87 |
| Dic | 250 | 0 | 95.14 | 2.57 |
| Linguistic | 250 | 0 | 78.46 | 4.43 |
| function | 250 | 0 | 63.47 | 4.31 |
| pronoun | 250 | 0 | 22.27 | 3.76 |
| ppron | 250 | 0 | 17.04 | 3.76 |
| i | 250 | 0 | 10.00 | 3.64 |
| we | 250 | 0 | 0.70 | 1.10 |
| you | 250 | 0 | 0.61 | 1.59 |
| shehe | 250 | 0 | 4.81 | 3.29 |
| they | 250 | 0 | 0.74 | 1.27 |
| ipron | 250 | 0 | 5.23 | 2.25 |
| det | 250 | 0 | 12.13 | 2.82 |
| article | 250 | 0 | 4.41 | 1.77 |
| number | 250 | 0 | 1.79 | 1.83 |
| prep | 250 | 0 | 12.61 | 2.82 |
| auxverb | 250 | 0 | 11.03 | 2.91 |
| adverb | 250 | 0 | 7.07 | 2.79 |
| conj | 250 | 0 | 8.11 | 2.38 |
| negate | 250 | 0 | 2.40 | 1.51 |
| verb | 250 | 0 | 21.41 | 3.88 |
| adj | 250 | 0 | 4.94 | 2.14 |
| quantity | 250 | 0 | 3.38 | 2.10 |
| Drives | 250 | 0 | 5.12 | 2.34 |
| affiliation | 250 | 0 | 2.25 | 1.85 |
| achieve | 250 | 0 | 0.71 | 0.69 |
| power | 250 | 0 | 2.18 | 1.66 |
| Cognition | 250 | 0 | 14.64 | 4.45 |
| allnone | 250 | 0 | 1.51 | 1.29 |
| cogproc | 250 | 0 | 13.05 | 4.29 |
| insight | 250 | 0 | 3.08 | 2.13 |
| cause | 250 | 0 | 1.97 | 1.76 |
| discrep | 250 | 0 | 2.26 | 1.54 |
| tentat | 250 | 0 | 2.57 | 1.87 |
| certitude | 250 | 0 | 0.61 | 0.72 |
| differ | 250 | 0 | 3.98 | 1.81 |
| memory | 250 | 0 | 0.11 | 0.39 |
| Affect | 250 | 0 | 6.51 | 3.31 |
| tone_pos | 250 | 0 | 2.08 | 1.67 |
| tone_neg | 250 | 0 | 4.11 | 2.88 |
| emotion | 250 | 0 | 2.45 | 1.95 |
| emo_pos | 250 | 0 | 0.59 | 0.96 |
| emo_neg | 250 | 0 | 1.65 | 1.57 |
| emo_anx | 250 | 0 | 0.39 | 0.66 |
| emo_anger | 250 | 0 | 0.38 | 0.70 |
| emo_sad | 250 | 0 | 0.24 | 0.48 |
| swear | 250 | 0 | 0.14 | 0.32 |
| Social | 250 | 0 | 14.72 | 4.50 |
| socbehav | 250 | 0 | 4.87 | 2.42 |
| prosocial | 250 | 0 | 0.81 | 0.97 |
| polite | 250 | 0 | 0.35 | 1.46 |
| conflict | 250 | 0 | 0.69 | 0.76 |
| moral | 250 | 0 | 0.30 | 0.51 |
| comm | 250 | 0 | 2.32 | 1.60 |
| socrefs | 250 | 0 | 9.74 | 3.67 |
| family | 250 | 0 | 0.93 | 1.32 |
| friend | 250 | 0 | 0.24 | 0.48 |
| female | 250 | 0 | 1.10 | 2.22 |
| male | 250 | 0 | 4.48 | 3.16 |
| Culture | 250 | 0 | 0.27 | 0.60 |
| politic | 250 | 0 | 0.01 | 0.07 |
| ethnicity | 250 | 0 | 0.00 | 0.03 |
| tech | 250 | 0 | 0.26 | 0.59 |
| Lifestyle | 250 | 0 | 1.74 | 1.62 |
| leisure | 250 | 0 | 0.15 | 0.34 |
| home | 250 | 0 | 0.42 | 0.62 |
| work | 250 | 0 | 0.80 | 1.04 |
| money | 250 | 0 | 0.37 | 0.80 |
| relig | 250 | 0 | 0.08 | 0.23 |
| Physical | 250 | 0 | 2.36 | 2.11 |
| health | 250 | 0 | 0.94 | 1.17 |
| illness | 250 | 0 | 0.14 | 0.35 |
| wellness | 250 | 0 | 0.07 | 0.33 |
| mental | 250 | 0 | 0.26 | 0.67 |
| substances | 250 | 0 | 0.06 | 0.24 |
| sexual | 250 | 0 | 0.15 | 0.46 |
| food | 250 | 0 | 0.16 | 0.39 |
| death | 250 | 0 | 0.11 | 0.27 |
| need | 250 | 0 | 0.58 | 0.86 |
| want | 250 | 0 | 0.54 | 0.87 |
| acquire | 250 | 0 | 1.12 | 1.15 |
| lack | 250 | 0 | 0.14 | 0.42 |
| fulfill | 250 | 0 | 0.11 | 0.26 |
| fatigue | 250 | 0 | 0.06 | 0.19 |
| reward | 250 | 0 | 0.03 | 0.19 |
| risk | 250 | 0 | 0.46 | 0.67 |
| curiosity | 250 | 0 | 0.20 | 0.55 |
| allure | 250 | 0 | 7.48 | 2.59 |
| Perception | 250 | 0 | 8.80 | 3.10 |
| attention | 250 | 0 | 0.28 | 0.57 |
| motion | 250 | 0 | 1.89 | 1.39 |
| space | 250 | 0 | 5.40 | 2.45 |
| visual | 250 | 0 | 0.49 | 0.81 |
| auditory | 250 | 0 | 0.30 | 0.68 |
| feeling | 250 | 0 | 0.89 | 1.13 |
| time | 250 | 0 | 5.28 | 2.35 |
| focuspast | 250 | 0 | 5.68 | 3.18 |
| focuspresent | 250 | 0 | 6.52 | 3.04 |
| focusfuture | 250 | 0 | 1.49 | 1.45 |
| Conversation | 250 | 0 | 0.48 | 0.85 |
| netspeak | 250 | 0 | 0.38 | 0.79 |
| assent | 250 | 0 | 0.10 | 0.37 |
| nonflu | 250 | 0 | 0.01 | 0.08 |
| filler | 250 | 0 | 0.03 | 0.14 |
| AllPunc | 250 | 0 | 15.30 | 5.02 |
| Period | 250 | 0 | 5.92 | 2.73 |
| Comma | 250 | 0 | 2.94 | 2.38 |
| QMark | 250 | 0 | 0.96 | 1.69 |
| Exclam | 250 | 0 | 0.10 | 0.33 |
| Apostro | 250 | 0 | 3.31 | 2.26 |
| OtherP | 250 | 0 | 2.07 | 2.61 |
